# Yeast Particles for Encapsulation of Terpenes and Essential Oils

**DOI:** 10.3390/molecules28052273

**Published:** 2023-02-28

**Authors:** Ernesto R. Soto, Florentina Rus, Zeynep Mirza, Gary R. Ostroff

**Affiliations:** Program in Molecular Medicine, UMass Chan Medical School, 373 Plantation Street, Worcester, MA 01605, USA

**Keywords:** yeast particle, terpenes, essential oils, microencapsulation, sustained release, controlled release

## Abstract

Terpenes and essential oils are materials of great commercial use due to their broad spectra of antibacterial, antifungal, membrane permeation enhancement and antioxidant biological properties, as well as for their use as flavors and fragrances. Yeast particles (YPs) are 3–5 µm hollow and porous microspheres, a byproduct of some food-grade yeast (*Saccharomyces cerevisiae)* extract manufacturing processes, that have been used for the encapsulation of terpenes and essential oils with high payload loading capacity (up to 500% weight) and efficiency, providing stability and sustained-release properties. This review focuses on encapsulation approaches for the preparation of YP–terpene and essential oil materials that have a wide range of potential agricultural, food and pharmaceutical applications.

## 1. Introduction

Essential oils are concentrated, complex mixtures of volatile and non-volatile compounds obtained from plants. Terpenes are organic compounds that constitute the main component of essential oils and can be obtained as highly purified (>90%) materials extracted from natural sources or that are chemically synthesized. Both essential oils and pure terpenes are of great commercial interest due to their broad range of functional properties. These materials are used as permeation enhancers and antioxidants in cosmetics, as flavors in foods or as additives in food packaging to prevent microbial spoilage oxidation, as naturally derived pesticides in agricultural products, and as bioactive compounds or excipients in nutraceuticals and pharmaceuticals [1,2,3,4,5]. The interest in the use of terpenes will continue increasing in response to consumer trends favoring the use of natural compounds in food, agricultural, consumer, and pharmaceutical products. 

However, despite their many applications, the use of terpenes and essential oils in commercial products presents a few challenges due to terpene’s low water solubility, volatility, and chemical decomposition when exposed to air, heat, light and moisture. There are also challenges in some applications due to safety limits and the need for targeted sustained delivery of the active compounds or marked organoleptic effects when used as food additives. These challenges are generally overcome by encapsulation of terpenes or essential oil compositions in delivery systems. Encapsulation improves the stability of terpene by reducing loss of terpene content due to volatility or by chemical degradation. It also masks undesirable flavors associated with some terpenes utilized as additives to prevent food spoilage and increases shelf-life storage, and depending on the encapsulation material, it can provide for sustained terpene release required in some applications such as agricultural and pharmaceutical products [6,7,8,9,10].

Encapsulation techniques commonly used in the preparation of terpene formulations include emulsification, spray drying, liposomes, molecular complexation, complex coacervation and nanoparticles [11,12,13,14,15,16,17,18,19,20,21,22,23,24,25,26,27,28,29,30,31,32,33,34,35,36]. An effective terpene encapsulation process should produce terpene compositions with (1) high payload encapsulation efficiency, (2) high terpene loading capacity, (3) homogenous payload distribution in the matrix carrier, (4) improved terpene protection, (5) retention of the biological activity of terpenes, and (6) sustained terpene release characteristics. In addition to these properties, the process of encapsulation should be inexpensive, work under mild processing conditions, and the preferred materials for terpene encapsulation should have mechanical strength, controlled release properties, and should not pose any safety risk. Examples of common encapsulation techniques currently used in the formulation of terpene products are presented in Table 1.

We have developed alternative methods using yeast particles (YPs) to efficiently encapsulate high levels of terpenes and essential oils. In this review, we present these different approaches to produce YP–terpene compositions with higher terpene payload capacity and encapsulation efficiency as well as and the methods to achieve controlled release.

## 2. Yeast Particles

Yeast particles (YPs) are 3–5 µm hollow and porous microspheres, a byproduct of some food-grade Baker’s yeast (*Saccharomyces cerevisiae*) extract manufacturing processes. The hollow cavity of YPs can be used for encapsulation of a broad range of hydrophilic and hydrophobic macromolecules and small molecules [37,38,39,40,41,42,43,44,45]. YPs offer several potential advantages as delivery carriers, such as high payload capacity, payload protection from external environmental stresses, possibility of controlled payload release, biocompatibility, and biodegradability. 

Some work in the literature describing the use of YPs for encapsulation of bioactives has reported a few challenges using YPs due to the difficulty of loading bioactive substances, resulting in low encapsulation efficiency, low payload capacity, and aggregation of payload on the outer surface YP wall rather than efficiently encapsulated within the hollow cavity of YPs [46,47,48,49,50]. These encapsulation challenges are significantly diminished by careful control of terpene:water:YP weight ratios during the loading process to maximize terpene diffusion into YPs. In this review, we report different methods for the encapsulation of terpenes and essential oils in YPs that result in the production of YP–terpene formulations with high payload capacity (up to 5:1 *w*/*w* terpene:YP ratio), high encapsulation efficiency (>95%), homogenous terpene loading inside the hollow cavity of YPs, and sustained terpene release.

## 3. First Generation YP–Terpenes—Diffusion-Based Loading and Release

**Figure 1:**
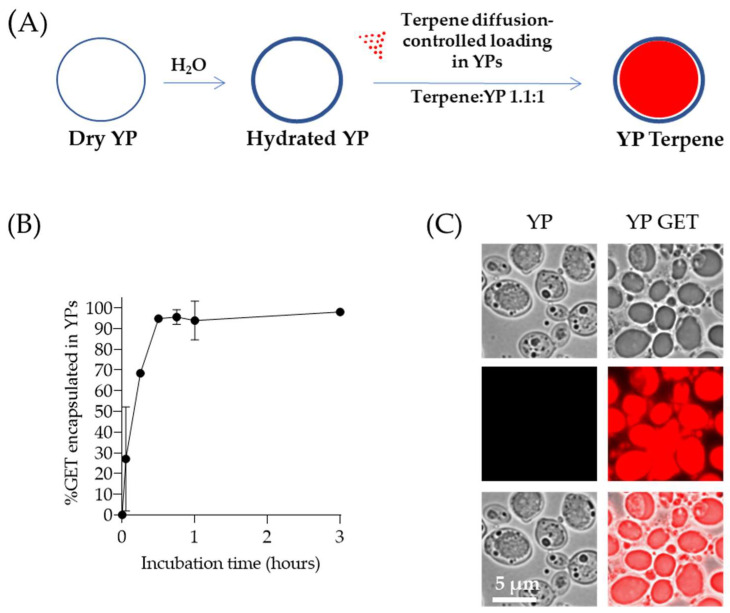
(**A**) Schematics of diffusion-controlled terpene loading in YPs, (**B**) kinetics of 2:1:2 GET loading in YPs quantified by HPLC of samples prepared at a GET:YP ratio of 1.1:1 in a homogenized YP suspension (final concentration of 150 g YP/L, 16.5% GET), and (**C**) microscopy images of Nile red-stained YP control (t = 0) and YPs loaded with GET after 1-h incubation. Brightfield images show a droplet inside YP GET, and Nile red staining of hydrophobic terpene confirms this droplet corresponds to a terpene droplet loaded in YPs. Figures adapted from Reference [40] and reproduced with permission from MDPI.

Terpene encapsulation in YPs is based on the loading of terpenes inside the hydrophobic YP cavity by the passive diffusion of terpenes through the porous cell walls in a homogenized aqueous suspension of YPs, as illustrated in Figure 1A. This YP–terpene loading method is a low-cost, easily scaled approach that produces stable YP–terpene suspensions up to a 2:1 *w*/*w* terpene:YP ratio, without the use of surfactants or alcohols to stabilize the terpene in the formulation. A mixture of geraniol (G), eugenol (E), and thymol (T) at a composition of 2:1:2 G:E:T weight ratio was used to evaluate the production of YP–terpenes. This GET mixture has been shown to be highly effective in agricultural applications against a broad range of plant pathogens [51]. The passive diffusion of the GET mixture into YPs in a homogenized aqueous YP suspension is a rapid process, and >95% of the GET mixture is encapsulated within one hour in samples prepared at a GET:YP weight ratio of 1.1:1 as shown by HPLC quantification in Figure 1B. The loading of terpenes inside the hollow cavity of YPs can be visualized by microscopy as a clear terpene droplet in the brightfield images and by using Nile red dye to stain terpenes as shown in Figure 1C. Nile red is a lipophilic stain; it does not fluoresce in a polar (water) solvent, but it is highly fluorescent in a hydrophobic (terpene) environment. These formulations are prepared and stored as homogenized stable, aqueous YP GET suspensions at 150 g YP/L, 165 g GET/L. At these high concentrations, the terpenes remain >95% encapsulated within the hollow hydrophobic cavity of the particles. Sustained terpene release is the reverse diffusion process that occurs upon sample dilution and is a function of terpene water solubility, which can be modified by the addition of surfactants or water-miscible organic solvents.

This first-generation YP–terpene encapsulation approach has been effectively implemented to develop and commercialize YP–terpene-based fungicide and nematicide products for agricultural applications [51,52,53,54,55,56]. This approach has also been used in the encapsulation of essential oils and fragrances to develop YP-based odor neutralizers, head-lice lotions, and household insecticidal sprays effective against fleas, dust mites, and bed bugs. 

In addition to the development of YP–terpenes for agricultural and consumer care products, we have evaluated YP–terpenes in studies focused on the potential development of YP–terpene formulations for pharmaceutical applications, specifically in the possible use of YP–terpenes for the treatment of gastrointestinal parasitic nematode (e.g., roundworm, hookworm, whipworm) infections. Terpenes have a long history of use as anthelmintics, and thymol was successfully employed to eradicate hookworm infections in the United States in the early 20th century [57]. Although effective, the medical application of thymol and other terpenes has long been discontinued due to the need of using large doses of terpenes, as >90% of orally administered terpene is rapidly absorbed in the stomach [58], resulting in toxic side effects and less than 10% of terpenes reaching the target sites in the intestine where parasitic nematodes reside. Currently, parasitic nematode infections affect ~1 billion people in developing countries [59], and the current anthelmintic drugs (benzimidazoles) employed in mass drug administration programs do not have broad specificity, and their application is at risk by the emergence of drug-resistant nematodes [60,61].

The encapsulation method developed for YP GET formulations was evaluated with 17 commercially available terpenes and three essential oils (lavender, tea tree, and peppermint oil) to produce YP–terpene (or essential oil) compositions at 1.1:1 *w*/*w* terpene:YP ratio [38]. All YP–terpene samples were produced with >95% encapsulation efficiency. These YP–terpenes were evaluated for their in vitro anthelmintic activity on hookworms (*Ancylostoma ceylanicum*, *Nippostronglyus brasiliensis*) and whipworms (*Trichuris muris*). The results showed that YP–terpenes are broad-acting anthelmintics [38]. The use of YPs as a terpene delivery carrier is a promising approach for the development of low-cost anthelmintics with broad specificity and with the potential to overcome parasite resistance.

Terpene release from this first-generation YP–terpene compounds is based on passive diffusion of terpenes through the porous YP cell walls and is a function of terpene water solubility. This release process can pose limitations in the application of YP–terpene materials, for example, for agricultural applications (rapid elution of terpenes from YP–terpene in diluted samples) or in the development of YP–terpenes as anthelmintics (dilution in the digestive tract leading to burst release before payload reaching target pathogens in the intestine). The following sections describe encapsulation approaches to produce YP–terpene formulations with better control of terpene release.

## 4. YP Encapsulation Methods to Extend Duration of Terpene Release

**Figure 2:**
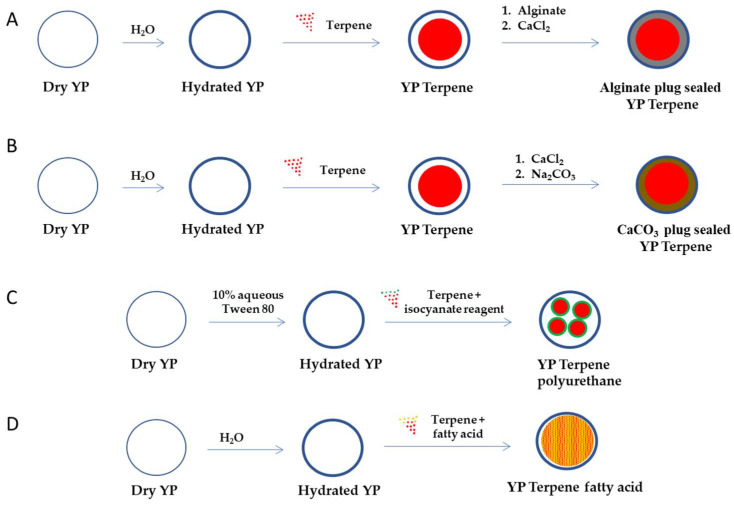
Schematic representation of steps required to encapsulate terpenes in YPs using (**A**) an alginate plug seal, (**B**) calcium carbonate plug seal, (**C**) encapsulation of terpenes in polyurethane nanoparticles, and (**D**) co-encapsulation with fatty acids (YP–terpene fatty acid can also be prepared by sequential loading of fatty acid and then the terpene).

To develop YP–terpene formulations with better control of terpene release, we evaluated alternative YP encapsulation strategies that prevent rapid burst release of terpene upon dilution of YP–terpenes below the solubility of the encapsulated terpene in water. These YP–terpene encapsulation approaches incorporate some aspects of other methods for terpene delivery and include the plug seal of YP–terpene samples using hydrocolloid crosslinked gels, encapsulation of terpenes in nanoparticles generated in situ in YPs, and co-loading of terpene with a hydrophobic sequestering agent. The steps to prepare these materials are schematically depicted in Figure 2.

### 4.1. YP–Terpene with a Hydrocolloid Plug Seal

Hydrogels are high-water content materials prepared by crosslinking polymers and have applications in encapsulation technologies due to their ability to trap and slowly release the target payload [62,63]. Several non-toxic, biocompatible, and biodegradable compounds such as the natural polysaccharides chitosan and alginate have been extensively studied in drug encapsulation. The use of hydrocolloids can be applied in YPs by loading of the hydrocolloid polymer precursor and crosslinking to form a shell embedding the payload and plug sealing the pores of YPs. This approach has been previously demonstrated by plug-sealing yeast particles encapsulating the TB drug rifampicin using alginate–calcium and chitosan hydrocolloids [64].

Hydrocolloid plug-sealed YP–terpene samples have been prepared using calcium crosslinked alginate. Alginate plug-sealed YP–terpene samples extend the duration of terpene release for a few hours compared to YP–terpenes. Limitations of this approach are the need for a rapid crosslinking reaction to plug seal the YP pores and prevent dissolution of the polymer matrix in the terpene payload.italics

### 4.2. YP–Terpenes with a Calcium Carbonate Plug Seal

The use of inorganic amorphous insoluble materials prepared in situ in YPs is an approach, similar to the use of hydrocolloids in which the YP pores are plug sealed by the inorganic matrix slowing diffusion of terpene release. Calcium carbonate can be formed in situ in YPs by absorption of calcium chloride and subsequent reaction with sodium carbonate or sodium bicarbonate at pH 9–10. This encapsulation method also extends the duration of terpene release for a few hours compared to YP–terpenes. The short extension of terpene release with hydrocolloid or carbonate plug seal methods over the first-generation YP–terpene technology limits their application. Encapsulation approaches with better control of payload release and that significantly extend the duration of terpene release from the particles are described in the following sections.

### 4.3. YP In Situ Encapsulation of Terpenes in Polyurethane/Polyurea Nanoparticles

We have previously shown that nanoparticles can be used in combination with YPs by either physisorption or chemical absorption by non-covalent or covalent binding of large nanoparticles (average diameter > 40 nm) to the outer surface of YPs, encapsulation of small nanoparticles (average diameter < 30 nm) inside the hollow cavity of YPs, or by in situ formation of nanoparticles in YPs [43,45,65]. Polyurethane/polyurea nanoparticle drug delivery systems are formed by the reaction of isocyanates and diols on the surface of an emulsion containing target drug [66,67].

YP–terpene polyurethane nanoparticles can be formed in situ in YPs by co-loading of terpene with the polyol/polyamine precursor and then crosslinking with an isocyanate reagent (e.g., isophorone diisocyanate, toluene diisocyanate).

**Figure 3:**
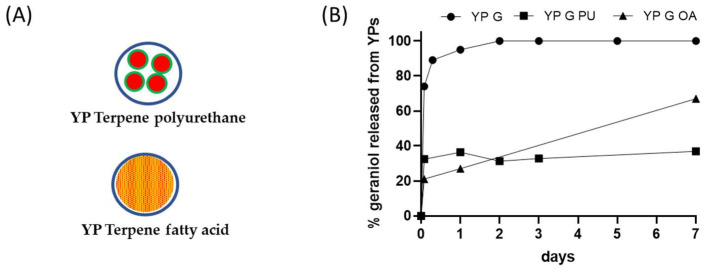
(**A**) Schematics of YP–terpene formulations prepared by encapsulation of terpene in polyurethane or co-loading of terpene with a fatty acid, and (**B**) kinetics of geraniol release from YP samples at 25 °C in YP–terpene suspensions at total geraniol concentration of 1 mg/mL (YP G: YP geraniol, YP G PU: YP geraniol polyurethane, YP G OA: YP geraniol oleic acid).

This YP–terpene encapsulation approach achieves high encapsulation efficiency, similar to YP–terpenes 1.1:1, and significantly decreases the rate of terpene release (Figure 3B). However, the approach has limitations due to the need to use toxic isocyanate crosslinkers, possible side crosslinking reactions of polyurethane with terpenes containing hydroxyl groups, and regulatory concerns with the application of polyurethane materials in agricultural products or for direct human health applications. However, this YP–terpene encapsulation approach has potential if developed using green materials such as non-isocyanate polyurethane nanoparticles [68]. 

### 4.4. YP Lipid Terpenes

Fatty acids can be used as sequestering agents of terpenes inside YPs. The rationale in this approach is to use fatty acids that (1) dissolve the target terpene compound and (2) are more hydrophobic than the terpene. The materials can be co-loaded in a single step or sequentially loaded in YPs. This approach generally requires loading of fatty acid and terpenes at a >1:1 fatty acid:terpene weight ratio to maximize terpene retention in the hydrophobic lipid environment. The selection of lipids with melting point above room temperature improves the stability of the YP–terpene lipid material by forming a solid matrix inside the cavity of YPs. The results in Figure 3B show significant reduction in the release of geraniol from a YP sample containing geraniol and oleic acid (1:1 oleic acid:geraniol) compared to the YP geraniol control. The geraniol release from YP–geraniol–oleic acid shows a burst release (~30% geraniol) between 0 and 4 h, and the remaining geraniol remains stably encapsulated up to 7 days (Figure 2B). These YP lipid terpene materials have potential use for long-term sustained terpene-release applications.

### 4.5. Hyper-Loaded YP–Terpenes 

**Figure 4:**
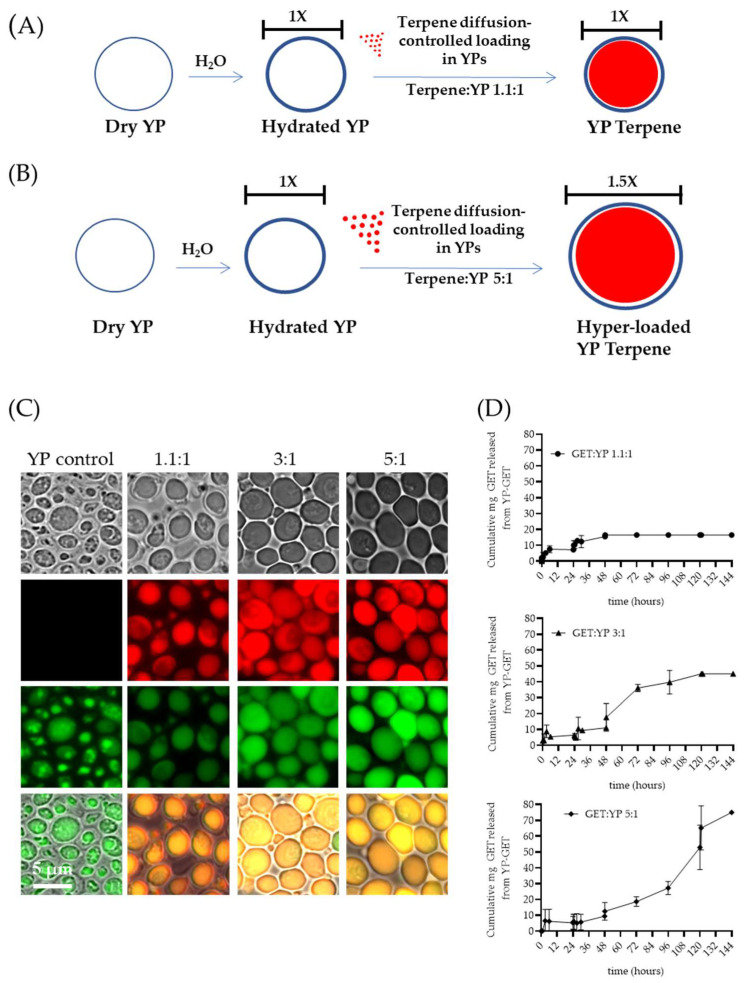
Schematics of diffusion-controlled terpene loading in YPs at terpene:YP ratios of (**A**) 1.1:1 and (**B**) 5:1. (**C**) Microscopy images showing FITC–concanavalin A stained YPs and Nile red stained terpenes in empty YP control and YP GETs loaded at GET:YP ratios of 1.1:1, 3:1, and 5.1. (**D**) Cumulative GET release from YPs showing extension of wetting/terpene release cycles in YP GET 1.1:1 and hyper-loaded YP GET at ratios of 3:1 and 5:1 GET:YP. Figures adapted from Reference [40] and reproduced with permission from MDPI.

The first-generation YP–terpene formulations were developed to encapsulate up to a 2:1 *w*/*w* terpene:YP ratio. Recently, methods to increase terpene loading capacity in YPs up to 5:1 *w*/*w* were reported in the literature [40]. The preparation of hyper-loaded YP–terpenes is possible by carefully controlling the ratio of water to YP to minimally hydrate the porous shell, allowing for slow diffusion of terpene payload. The hyper-loading of terpenes in YPs leads to an increase in YP diameter from 5.4 µm (empty YPs) to 7.7 µm (YP–terpene 5:1) to accommodate the large terpene droplet at higher payload levels in the swollen hollow cavity of the particles (Figure 4A–C). In addition to increasing efficient payload loading capacity, these hyper-loaded YP–terpene samples provide for enhanced sustained terpene release, up to three-fold compared to the previously developed YP–terpene 1.1:1 formulation (Figure 4D) and show higher antimicrobial potency than unencapsulated terpenes. The original work also described methods to improve thermal terpene storage encapsulation stability by producing hyper-loaded YP–terpene samples in a solvent mixture of 70% water–30% glycerin at 100 g YP/L and water-suspended terpene concentrations from 300 to 450 g/L.

**Figure 5:**
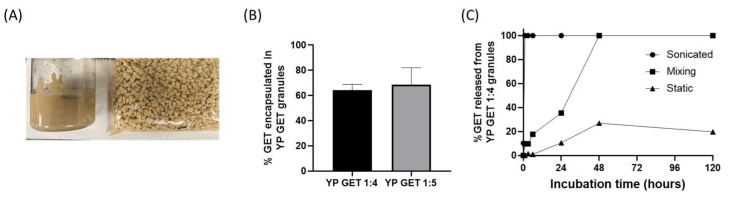
(**A**) Picture of homogenized aqueous suspension of YP GET 1:4 and YP GET 1:4 granules, (**B**) encapsulation efficiency of GET 2:1:2 in YP GET granules, and (**C**) GET release from YP GET 1:4 granules suspended in water at 1 mg GET/mL at room temperature under three incubation conditions.

The hyper-loaded YP–terpenes previously reported in the literature were prepared as homogenized liquid suspensions. It is also possible to extrude the hyper-loaded YP–terpene suspension to produce dry hyper-loaded YP–terpene granules (Figure 5A). Samples of YP GET suspensions hyper-loaded at GET:YP weight ratios of 4:1 and 5:1 were extruded to form YP GET granules. The dry granules were characterized for encapsulation efficiency and kinetics of terpene release. The results show that ~60–70% of GET was retained inside YPs during the extrusion and drying steps (Figure 5B). The kinetics of release from YP GET 1:5 granules was evaluated under three conditions: (1) granules that were suspended in water and sonicated, resulting in rapid disaggregation of granules into single YPs, (2) granules suspended in water and mixed with gentle agitation, and (3) granules suspended in water and not mixed (static). The results in Figure 4C show the effect of mixing on the release of GET from YP GET granules, samples that were sonicated showed burst GET release, YP GET granules that were mixed with mild agitation released payload in ~48 h, and samples that were not mixed only released up to 20% GET after 5 days of incubation in water at a target GET concentration of 1 mg/mL.

The development of hyper-loaded YP–terpenes has a wide range of potential agricultural and pharmaceutical applications that could benefit from a delivery system with a high payload loading capacity combined with increased payload stability and sustained release properties.

### 4.6. YP–Terpene with a pH-Sensitive Eudragit^®^ Coating

Eudragit^®^ is a brand name of polymethacrylate-based copolymers. These copolymers are composed of methacrylic acid and methacrylic/acrylic esters and are modified to include anionic, cationic and/or neutral charge. The polymers are responsive under different pH conditions and are extensively used in pharmaceutical coatings for taste-masking and targeted delivery [69,70].

**Figure 6:**
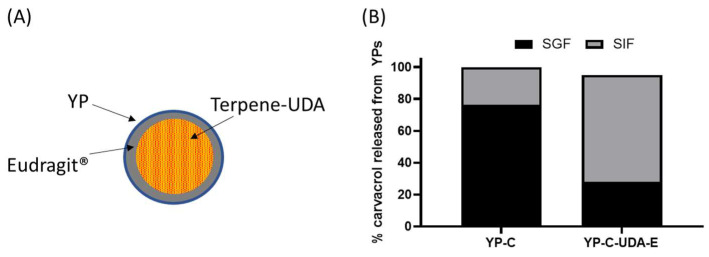
(**A**) Schematic of a YP–terpene–UDA particle coated with Eudragit^®^ polymer and (**B**) carvacrol released from YPs in YP carvacrol (YP-C) control sample and YP carvacrol co-loaded with UDA and coated with Eudragit^®^ L-100 (YP-C-UDA-E). The release assay was performed by sequential incubation of YP samples in simulated gastric fluid (SGF) at 37 °C for 2 h followed by incubation in simulated intestinal fluid (SIF) at 37 °C for 2 h. Carvacrol concentration during simulated digestion was 1 mg/mL.

We evaluated the coating of YP carvacrol with Eudragit^®^ L-100, a polymer that forms an insoluble coat below pH < ~5.5 and dissolves at pH > 5.5 and is used in pharmaceutical coating of pills for oral delivery of drugs to the intestine. It was not possible to efficiently coat YP carvacrol or other terpenes with Eudragit^®^ L-100 or other Eudragit^®^ polymers, as the terpene dissolves the coat regardless of pH. This limitation was overcome by coating a mixture of carvacrol with undecanoic acid (UDA), as the fatty acid acts as a sequestering agent of carvacrol, preventing the terpene from dissolving the coat. Samples of YP carvacrol–UDA–Eudragit^®^ L-100 and YP carvacrol control were incubated in simulated digestion conditions: first, the samples were incubated in simulated gastric fluid (SGF, pH 1.5) with pepsin for 2 hours at 37 °C; the samples were centrifuged, and the SGF was collected for HPLC analysis of released carvacrol; the YP pellets were suspended in simulated intestinal fluid (SIF, pH 6.8) with pancreatin at 37 °C; and the SIF was also analyzed for released carvacrol after 2 h incubation. The results in Figure 6B show that coated samples were effective at reducing carvacrol release in SGF, and they released the payload in SIF after dissolution of the Eudragit^®^ coat. These results are encouraging, as this approach offers the possibility of selectively targeting terpenes to the intestine. However, this approach has the limitation that the use of fatty acid and Eudragit^®^ L-100 reduces the loading capacity of terpenes in YPs. 

### 4.7. YP Pro-Terpene with Stimuli-Controlled Terpene Release

**Figure 7:**
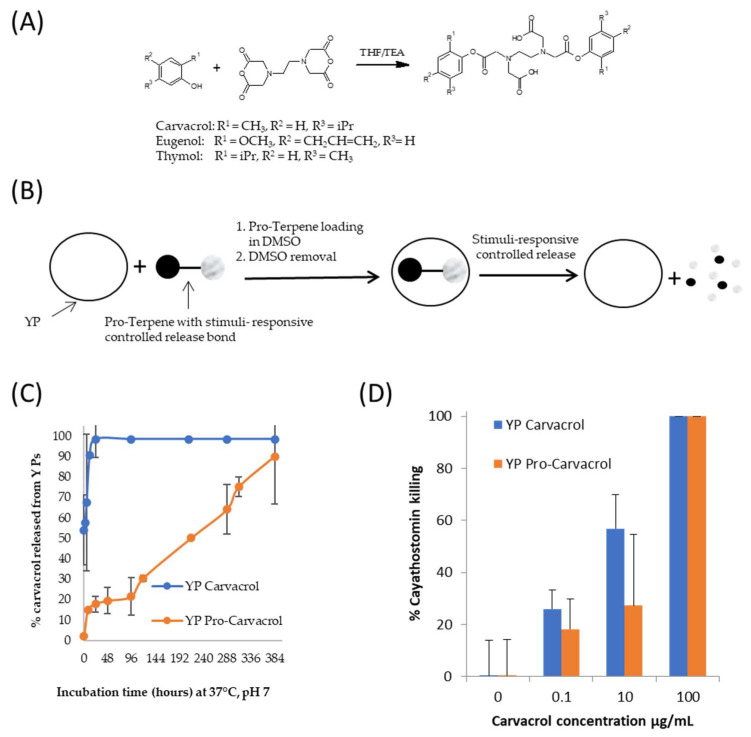
(**A**) Synthesis of pro-terpenes from parent terpene compounds (eugenol, carvacrol, thymol) and EDTA dianhydride, (**B**) schematics of pro-terpene loading in YPs and stimuli-controlled terpene release, (**C**) kinetics of carvacrol release from YP carvacrol and YP pro-carvacrol in 0.1 M phosphate buffer saline (PBS, pH 7) at 37 °C at a concentration of 1 mg carvacrol/mL, and (**D**) in vitro activity of carvacrol samples in cyathostomin egg-to-larvae assay. Figures adapted from Reference [39] and reproduced with permission from MDPI.

A method to use YP for delivery of terpenes with stimuli-responsive control of terpene release was recently developed [37,39]. Biodegradable pro-terpene compounds were synthesized (Figure 7A) and encapsulated in YPs (Figure 7B) at a target weight ratio of 1:1 terpene:YP (~1.7–1.9 pro-terpene:YP weight ratio). In one pro-carvacrol-YP example formulation (Figure 7C), the kinetics of carvacrol release at pH 7, 37 °C, in suspensions at 1 mg carvacrol/mL show that it takes up to 16 days for YP pro-carvacrol to completely release its carvacrol content. The YP carvacrol control completely released carvacrol in < 1 d. The kinetics of terpene release from YP pro-carvacrol increased at higher pH (complete release in < 2 h at pH 10) or at acidic or neutral pH in the presence of esterases that hydrolyze the scaffolded terpene compound. In addition to the stimuli-responsive release of terpenes, the YP pro-terpenes show higher encapsulation stability than YP–terpenes due to pro-terpenes being non-volatile solids at room temperature. 

The YP pro-terpenes retained the full biological activity of the parent terpene compound in antibacterial, antifungal, and anthelmintic assays. The results in Figure 7D are an example of retention of biological in vitro activity showing that YP pro-carvacrol had similar efficacy as YP carvacrol in a cyathostomin egg-to-larvae assay. These YP pro-terpene samples have potential for development in pharmaceutical applications due to their improved stability over YP–terpenes and stimuli-responsive controlled release. 

Yeast particles can be used for the encapsulation of terpenes and essential oils with high payload capacity and efficiency. The use of YPs allows for encapsulation levels up to 5:1 terpene:YP weight ratio; this high encapsulation capacity is achieved without the use of alcohols and potentially toxic surfactants typically required to stabilize terpenes in different formulations. The key attributes and limitations of the various terpene encapsulation approaches are summarized in Table 1. 

## 5. Summary and Future Perspectives

The broad applications for terpenes range from antioxidants, flavors and odors in foods and cosmetics, to biocides, insecticides, and pesticides in agricultural and consumer products, to bioactive compounds or permeation enhancing excipients in nutraceuticals and pharmaceuticals. These broad applications of terpenes as natural compounds in food, agricultural, consumer, and pharmaceutical products will continue to grow if the limitations of terpenes are addressed, by encapsulation of terpenes or essential oil compositions in appropriate delivery systems. Encapsulation improves the stability of terpene by reducing loss of terpene content due to volatility or by chemical degradation. It also masks undesirable flavors associated with some terpenes utilized as additives to prevent food spoilage, increases shelf-life storage, and depending on the encapsulation material, it can provide for sustained terpene release required in some applications such as agricultural and pharmaceutical products [6,7,8,9,10].

A number of different formulation strategies have been employed to overcome the limitations of terpenes. Most approaches only address some of these limitations of terpene’s poor water solubility, volatility, organoleptic properties and stability to air, heat, light and moisture, as summarized in Table 1.

We have developed the use of yeast particles for the encapsulation and delivery of terpenes and essential oils in the hollow cavity of YPs based on the passive diffusion of payloads through the porous yeast cell walls. YP–terpene compositions can be prepared with high loading capacity, up to 5:1 terpene:YP ratio (which is significantly higher than other available encapsulation methods for terpenes), high encapsulation efficiency, enhanced terpene stability, and sustained release properties. The first-generation approach to prepare YP–terpenes was successfully implemented to develop and commercialize YP–terpene-based fungicide and nematicide products for agricultural applications. Further advancement of YP-encapsulated terpenes has focused on developing materials with improved controlled of terpene release by methods that reduce the rate of diffusion-based release of terpene from YPs or the use of YP–terpene compositions with stimuli-response (pH, enzyme) release. The development of these different approaches for encapsulation of YP–terpenes has the potential of enabling the use of terpenes for a wide range of agricultural, food and pharmaceutical applications.

## Figures and Tables

**Table 1:** Encapsulation methods that can be used to prepare terpene and essential oil formulations.

Terpene Encapsulation Method	Advantages	Limitations	References *
Emulsification	Large-scale preparation possible using homogenization techniques	Requires use of alcohols and surfactants to stabilize terpene emulsions Low payload capacity (<25% *w*/*w* in double emulsion systems)	[15,16,17]
Nanoparticles	Different polymer reagents allow for production of particles with different compositions, sizes, controlled release	Limited payload loading capacity <50% terpene *w*/*w* Terpene loss in nanoparticle synthesis methods requiring heating or solvent evaporation steps	[18,19,20,21]
Liposomes	Increased stability within the hydrophobic inner core of liposome	Requires use of surfactants to stabilize terpene liposomes High production cost Liposome-based products have short half-life	[22,23]
Ionic gelation	Crosslinked microgels protect and control payload release	Limited payload loading capacity of 0.25–0.85 g terpene:weight ratio	[24,25,26,27]
Molecular complexation	Improves terpene water-solubility, thermal stability, reduced volatility, increased bioavailability	Limited payload loading capacity of <0.5 g terpene:weight ratio	[27,28,29,30,31]
Complex coacervation	High payload capacity and encapsulation efficiency Mild processing conditions	Lacks controlled release Complex coacervate capsules or shells lack stability and can become plasticized	[32,33]
Spray drying	Continuous process with short residence time (limited loss of payload) for formation of particles with control over particle size	Limited payload loading capacity of <0.5g terpene:weight ratio	[34,35,36]
**Yeast Particle (YP) terpenes** First generation Plug seal hydrocolloid Calcium carbonate Nanoparticles Lipid sequestration Enteric coating Pro-terpenes	High payload capacity (up to 5:1 terpene:weight ratio or 500% by weight) and encapsulation efficiency (>95%) Non-toxic compounds are used in preparation of formulation (exception polyurethane nanoparticles) Controlled release Long shelf-life: concentrated first-generation YP–terpene aqueous suspensions at 15% YP, 16.5% terpene remain stably encapsulated >5 years; YP pro-terpene approach uses non-volatile pro-terpene compounds Stable to light and oxidation	Particle settling, yeast taste, odor and tan color can impact the application at high active levels Addition of excipients lowers payload loading capacity	[37,38,39,40]

* Examples of recent publications are listed for each encapsulation method.

## Data Availability

Not applicable.
